# Polymeric hydrophilic ionic liquids used to modify magnetic nanoparticles for the highly selective enrichment of N-linked glycopeptides

**DOI:** 10.1038/s41598-017-07516-x

**Published:** 2017-08-01

**Authors:** Fenglong Jiao, Fangyuan Gao, Heping Wang, Yulin Deng, Yangjun Zhang, Xiaohong Qian, Yukui Zhang

**Affiliations:** 1 School of Life Science and Technology, Beijing Institute of Technology, Beijing, 100081 China; 2 State Key Laboratory of Proteomics, National Center for Protein Science Beijing, Beijing Institute of Radiation Medicine, Beijing, 102200 China; 3 National Chromatographic Research and Analysis Center, Dalian Institute of Chemical Physics, Chinese Academy of Sciences, Dalian, 116011 China

## Abstract

The low abundance of glycopeptides in biological samples makes it necessary to enrich them before further analysis. In this study, the polymeric hydrophilic ionic liquid-modified magnetic (Fe_3_O_4_@MPS@PMAC) nanoparticles were synthesized via a one-step reflux-precipitation polymerization. Owing to the excellent hydrophilicity and strong electrostatic interaction toward glycopeptides of the polymerized hydrophilic ionic liquid, [2-(methacryloyloxy) ethyl] trimethylammonium chloride (MAC), the synthesized Fe_3_O_4_@MPS@PMAC nanoparticles exhibited outstanding performance in glycopeptide enrichment with high detection sensitivity (10 fmol), large binding capacity (100 μg mg^−1^) and satisfied enrichment recovery (approximately 82%). Furthermore, the newly developed Fe_3_O_4_@MPS@PMAC nanoparticles were applied for the glycopeptide enrichment of HeLa exosome proteins. A total of 1274 glycopeptides from 536 glycoproteins were identified in three replicate analyses of 50 μg of HeLa exosome proteins. These results demonstrate the potential of Fe_3_O_4_@MPS@PMAC nanoparticles for both glycoproteomic analysis and exosome research.

## Introduction

Protein glycosylation is one of the most important post-translational modifications, which is closely related to a variety of biological processes such as cell division, signal transduction, protein-protein interactions and tumor immunology^1–5^. It is reported that abnormal protein glycosylation is involved in the occurrence of many diseases^6^. Therefore, the detection of glycoproteins and characterization of glycan structures is vital to the study of its biological functions and disease diagnosis. Recently, mass spectrometry-based techniques have been widely used for glycoprotein analysis^7, 8^. However, the low abundance of glycopeptides and low ionization efficiency make it difficult for the in-depth characterization of glycoprotein by mass spectrometry-based strategies. Therefore, it is necessary to develop efficient methods to enrich glycopeptides before MS analysis^9, 10^.

Therefore, several novel materials and methods have been reported for glycopeptide enrichment including boronic acid chemistry^11–13^, hydrazide chemistry^14–16^, lectin affinity chromatography^17, 18^ and hydrophilic interaction chromatography (HILIC)^19–21^. The advantages and disadvantages of each method have been reported. The boronic-acid modified materials were employed for the enrichment of N-link and O-link glycopeptides. However, the strict pH requirement limited the application of these materials. Hydrazide chemistry has been developed as a specific and efficient method for glycopeptide enrichment, but the cumbersome pre-treatment was always required. The lectin affinity chromatography could be utilized for glycopeptide and glycoprotein enrichment by the affinity interactions between lectin and glycans, while a single lectin could not be used for a global enrichment of multiple types of glycopeptides. Among these methods, hydrophilic interaction chromatography has drawn significant attention since it provides high selectivity and good reproducibility. The enrichment conditions are mild such that the enrichment process does not destroy the glycan structure. A variety of materials were reported as supports for the synthesis of HILIC matrices, such as cellulose^22–24^, magnetic nanoparticles^25–27^, graphene-oxide^28, 29^ and metal-organic frameworks^30, 31^. In the last decade, magnetic nanoparticles have showed great superiority in biomaterials synthesis due to their unique biocompatibility, strong magnetic response and large specific surface area. Surface-modified magnetic nanoparticles have been successfully used for glycopeptide enrichment, such as maltose-functionalized iron oxide magnetic nanoparticles^32^, PEG brush hybrid hydrophilic magnetic nanoparticles^33^ and zwitterionic polymer-coated core-shell magnetic nanoparticles^34^. These types of magnetic nanoparticles have exhibited high efficiencies, good selectivity and large binding capacities for glycopeptide enrichment.

In recent years, ionic liquids have gained attention due to their unique physicochemical properties, such as their excellent solubility and good thermal stability as well as being capable of tunable extraction of specific target analytes^35^. In addition, ionic liquids are organic salts consisting of organic cations and inorganic anions. The functional groups in these salts can be transformed into a variety of structures for specific purposes, such as solid-phase extraction^36, 37^, energy storage^38, 39^, and chromatographic separation^40, 41^, among other purposes. Although there are a growing number of studies on ionic liquids modified magnetic nanoparticles^42–44^, there has been no report of the utilization of these novel material composites for glycoproteomic research.

Herein, for the first time, we report a polymeric hydrophilic ionic liquid-modified magnetic (Fe_3_O_4_@MPS@PMAC) nanoparticle for the selective enrichment of glycopeptides via hydrophilic interaction and electrostatic interaction. The abundant grafted ionic liquids on the surface of the nanoparticles increased the enrichment efficiency and binding capacity. High selectivity, sensitivity, and recovery of the glycopeptide enrichment from standard glycoproteins were achieved due to the excellent hydrophilicity of MAC. In addition, exosomes have received extensive attention as an important biomarker resource in recent years. It has been reported that exosomes are relevant to the targeted metastasis of cancer cells which are associated with abnormal glycosylation^45^. Therefore, the newly developed material was further used to capture the glycopeptides in the tryptic digest of proteins extracted from HeLa cell exosomes. In total, 1274 N-glycopeptides from 536 glycoproteins were identified, suggesting a promising application of this method in both glycoproteomic analysis and exosome research.

## Results and Discussion

### Preparation of Fe_3_O_4_@MPS@PMAC nanoparticles

**Figure 1:**
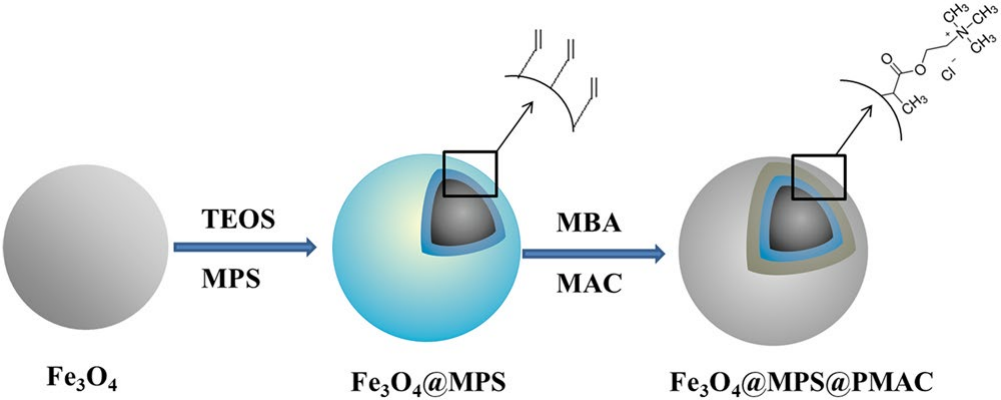
Schematic illustration of the synthetic procedure of Fe_3_O_4_@MPS@PMAC nanoparticles.

The fabrication procedure of Fe_3_O_4_@MPS@PMAC nanoparticles is shown in Fig. 1. Initially, Fe_3_O_4_ was synthesized via a solvothermal reaction and modified with TEOS to form a layer of SiO_2_. Then, MPS was loaded onto the Fe_3_O_4_ to provide double bonds for reflux-precipitation polymerization. Finally, the reflux-precipitation polymerization was carried out with MAC as monomer, MBA as the crosslinker, and AIBN as the initiator.

As a widely reported polymerization method, reflux-precipitation polymerization is rarely used for hydrophilic materials synthesis since many hydrophilic monomers are insoluble in commonly used reagents such as acetonitrile. To solve this problem, ionic liquid, MAC was induced as monomer in this study. As a commonly used monomer for free radical polymerization, MAC exhibited good solubility in common solvents such as acetonitrile, ethanol, DMF and so on. Meanwhile, combined with the advantages of excellent hydrophilicity and strong electrostatic interaction toward glycopeptides, MAC is quite suitable for reflux-precipitation polymerization of glycopeptides enrichment materials.

**Figure 2:**
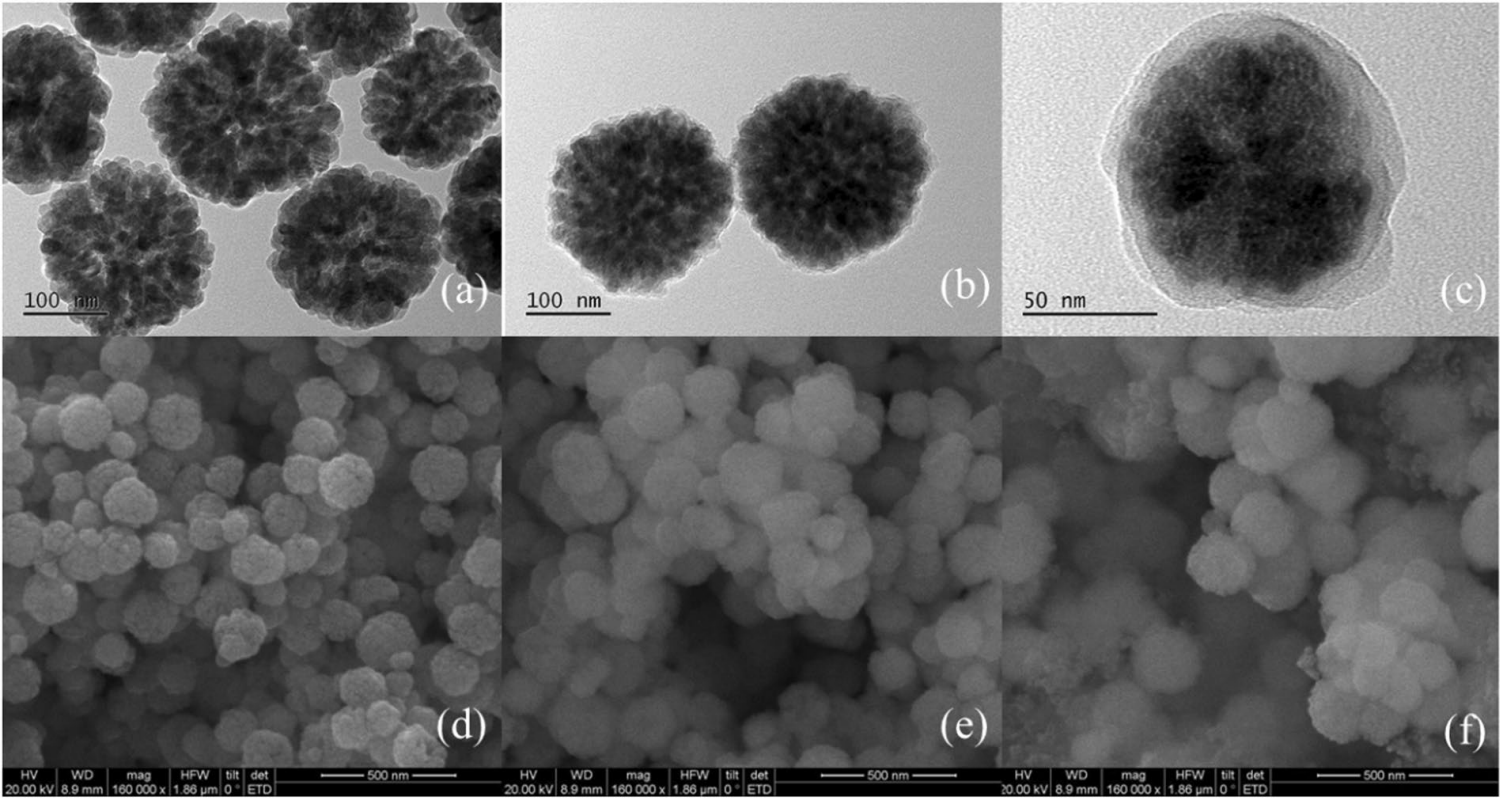
TEM images of (**a**) Fe_3_O_4_, (**b**) Fe_3_O_4_@MPS and (**c**) Fe_3_O_4_@MPS@PMAC. SEM images of (**d**) Fe_3_O_4_, (**e**) Fe_3_O_4_@MPS and (**f**) Fe_3_O_4_@MPS@PMAC.

SEM and TEM were employed to characterize the morphology of the synthesized Fe_3_O_4_@MPS@PMAC. As shown in Fig. 2, the Fe_3_O_4_ nanoparticles exhibit uniform surfaces with an average diameter of 150 nm. After polymerization, a MAC layer with an average thickness of 10 nm appeared and the nanoparticles agglomerated (Fig. 2f). Morphological changes from Fe_3_O_4_ to Fe_3_O_4_@MPS@PMAC demonstrated that the Fe_3_O_4_@MPS@PMAC nanoparticles were synthesized successfully.

**Figure 3:**
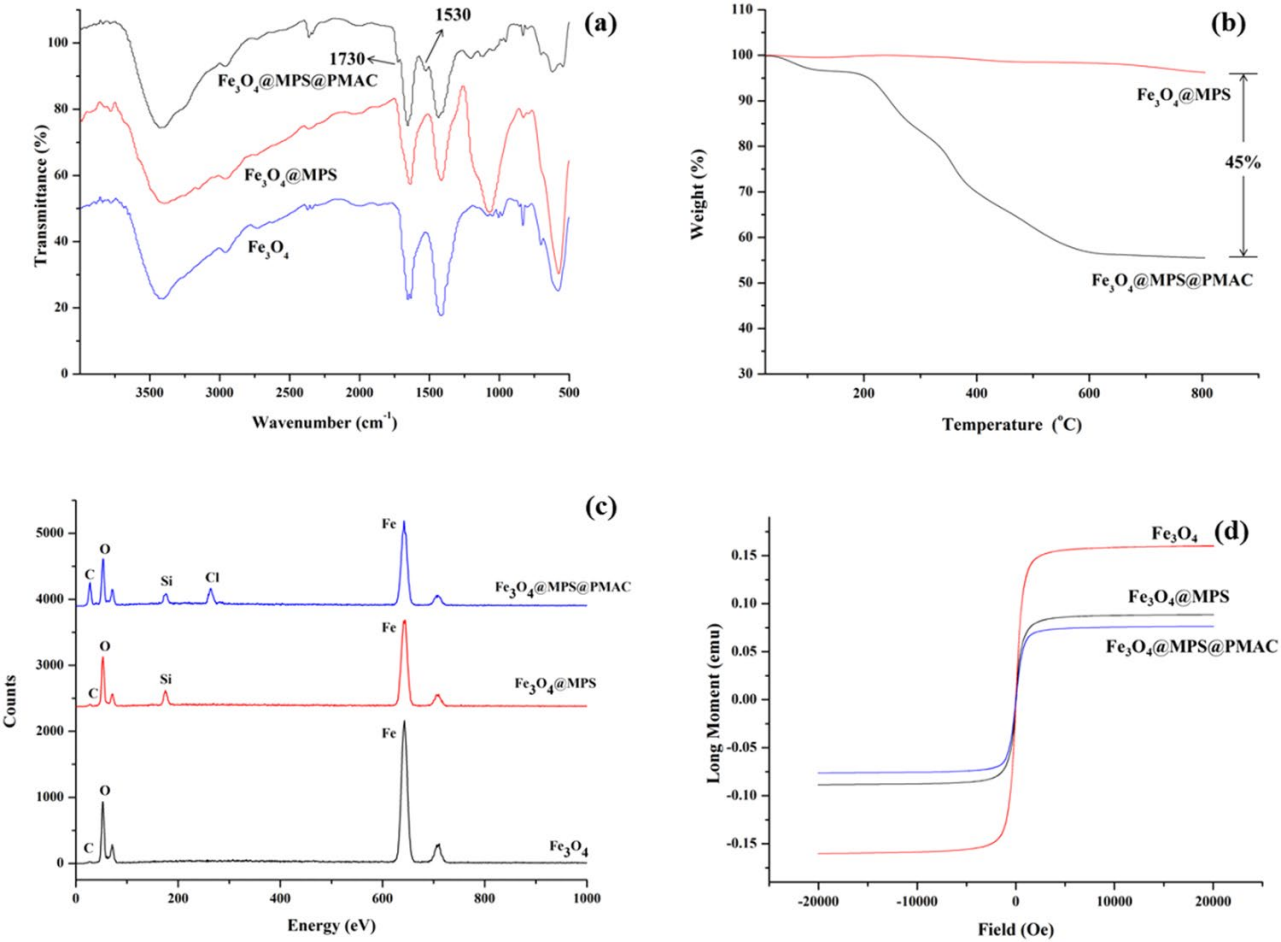
(**a**) FT-IR spectra, (**b**) TGA curves of Fe_3_O_4_@MPS and Fe_3_O_4_@MPS@PMAC, (**c**) Energy dispersive X-ray (EDX) spectrum and (**d**) Magnetization hysteresis loops of Fe_3_O_4_, Fe_3_O_4_@MPS and Fe_3_O_4_@MPS@PMAC.

The fabrication of Fe_3_O_4_@MPS@PMAC was also confirmed by FT-IR. As shown in Fig. 3a, an absorption at 1050 cm^−1^ not present in the FT-IR spectrum of Fe_3_O_4_ appeared in the spectrum of Fe_3_O_4_@MPS. The absorption was ascribed to the stretching vibration of Si-O-Si that revealed the successful grafting of TEOS and MPS. The absorptions at 1530 cm^−1^ and 1730 cm^−1^ in the spectrum of Fe_3_O_4_@MPS@PMAC corresponded to the bending vibration of N-H in MBA and C=O enhanced stretching vibration, respectively. The differences in the FT-IR spectrums of Fe_3_O_4_, Fe_3_O_4_@MPS and Fe_3_O_4_@MPS@PMAC proved that MAC was successfully coated onto the Fe_3_O_4_ nanoparticles.

Fe_3_O_4_@MPS@PMAC nanoparticles were further evaluated by TGA analysis, and the result is shown in Fig. 3b. Before grafting the MAC, the weight loss of Fe_3_O_4_@MPS was approximately 5%, which might be attributed to the coated MPS. For Fe_3_O_4_@MPS@PMAC, an additional weight loss of approximately 45% was observed. The obvious weight loss indicated that a large amount of MAC could be bound to the surface of the Fe_3_O_4_ nanoparticles by a one-step reflux-precipitation polymerization.

Energy-dispersive X-ray analysis (EDX) results are shown in Fig. 3c and Table S1. The content of Si and C in Fe_3_O_4_@MPS increased significantly compared with Fe_3_O_4_, indicating the successful modification with TEOS and MPS. The presence of Cl and increase of N in Fe_3_O_4_@MPS@PMAC demonstrated the successful polymerization of MAC on the magnetic nanoparticles.

The magnetic properties of Fe_3_O_4_@MPS@PMAC nanoparticles were tested using a vibrating sample magnetometer at room temperature. As shown in Fig. 3d, the final Fe_3_O_4_@MPS@PMAC nanoparticles exhibited a lower saturation magnetization value compared with that of the Fe_3_O_4_ and Fe_3_O_4_@MPS. The magnetic reduction of the Fe_3_O_4_@MPS@PMAC nanoparticles was due to the large amount of modified MAC. Although the magnetic properties decreased after modification, the Fe_3_O_4_@MPS@PMAC could still be easily separated by a magnet within 20 seconds.

### Glycopeptide enrichment from the tryptic digests of a standard glycoprotein

**Figure 4:**
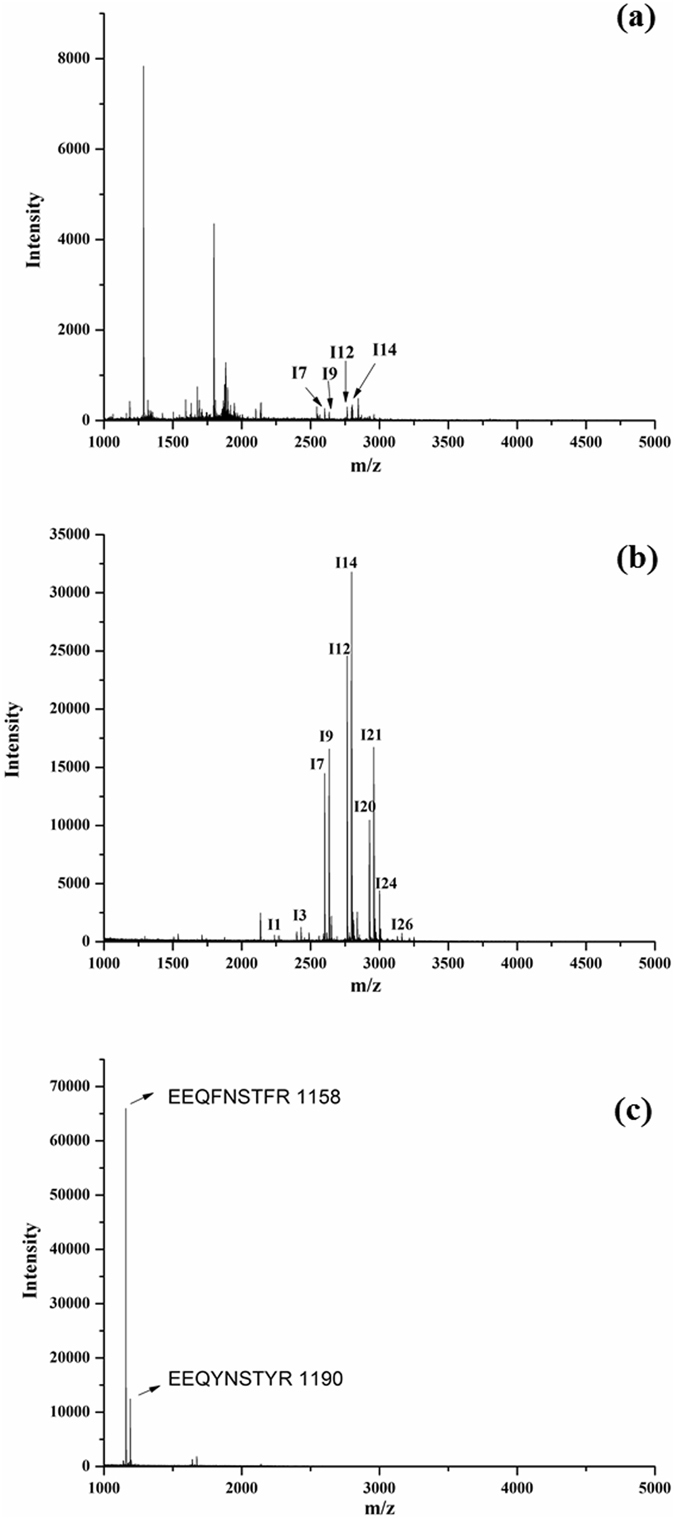
MALDI-TOF MS spectra of (**a**) direct analysis of 1 μg tryptic digest of human IgG; (**b**) after enrichment by Fe_3_O_4_@MPS@PMAC; (**c**) after enrichment by Fe_3_O_4_@MPS@PMAC and deglycosylation by PNGase F.

To evaluate the enrichment efficiency of Fe_3_O_4_@MPS@PMAC, a standard glycoprotein, human IgG, was used as a model. As shown in Fig. 4, for the direct analysis of the tryptic digests of human IgG, only 4 glycopeptide peaks were observed with weak peak intensity, while non-glycopeptides were detected with a much stronger signal (Fig. 4a). After enrichment with Fe_3_O_4_@MPS@PMAC, 27 glycopeptides could be identified with an obviously enhanced signal-to-noise ratio (Fig. 4b and Table S2) and non-glycopeptide signals were almost removed. Moreover, only two deamidated peptides (m/z = 1158 and 1190) could be observed after deglycosylated the eluted glycopeptides by PNGase F (Fig. 4c). These results further confirmed that the peaks identified in Fig. 4b were attributed to the glycopeptides.

**Figure 5:**
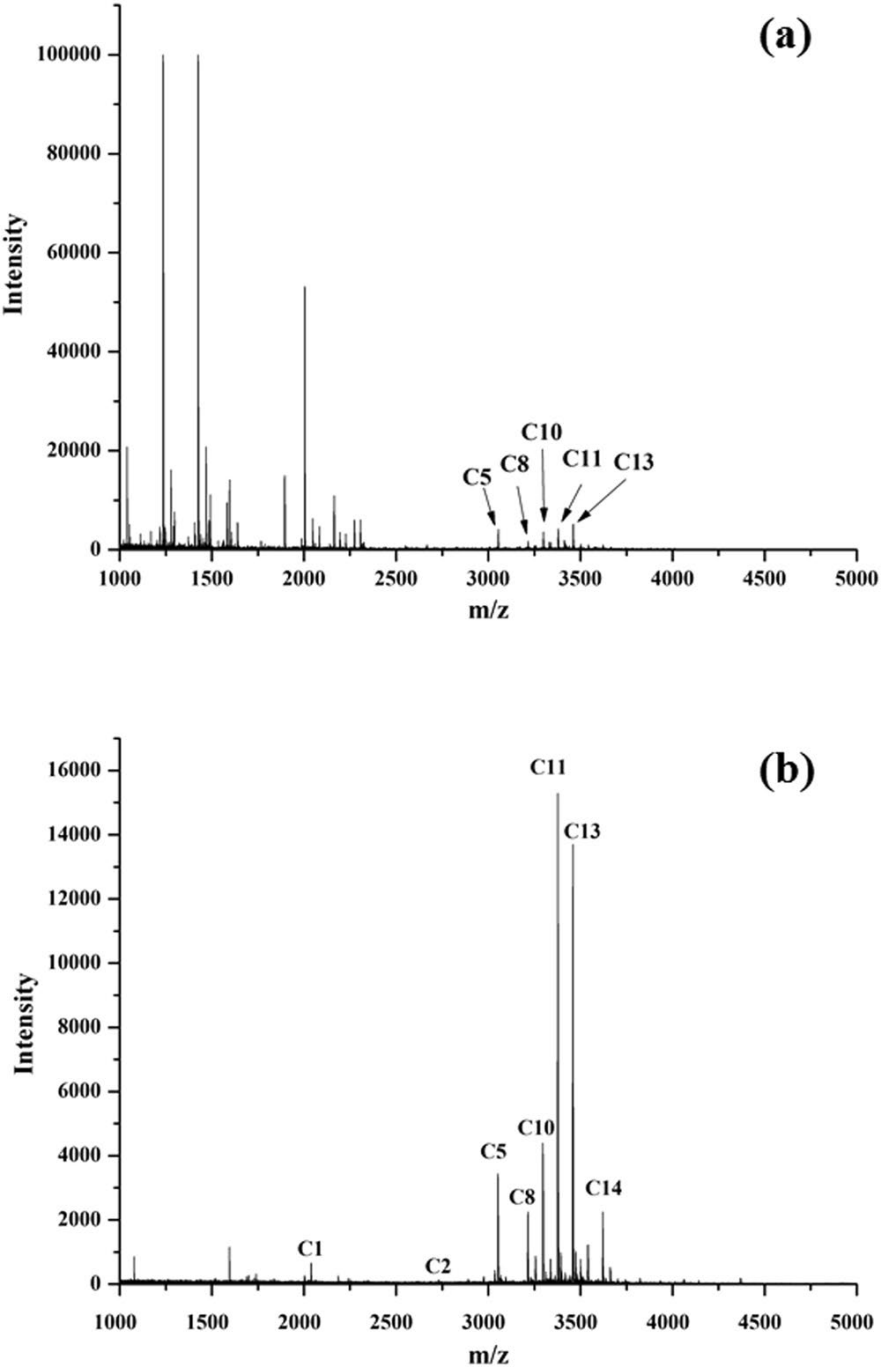
MALDI-TOF MS spectra of (**a**) direct analysis of 1 μg tryptic digest of CA and (**b**) after enrichment by Fe_3_O_4_@MPS@PMAC.

The tryptic digests of CA were used to further test the glycopeptide enrichment performance of Fe_3_O_4_@MPS@PMAC. The MALDI-TOF spectrum of direct tryptic digests of CA is shown in Fig. 5a. A similar result was observed with human IgG. Only 5 glycopeptide peaks with weak intensity were detected before enrichment, but a large number of non-glycopeptides were identified simultaneously with strong signals. After enrichment by Fe_3_O_4_@MPS@PMAC, 14 glycopeptides could be identified clearly and non-glycopeptides were almost removed (Fig. 5b and Table S3). These results further demonstrated the newly developed material can efficiently enrich glycopeptides. We attribute this good performance to the numerous anion and cation groups from modified MAC and its strong hydrophilicity for high selective enrichment of glycopeptides.

**Figure 6:**
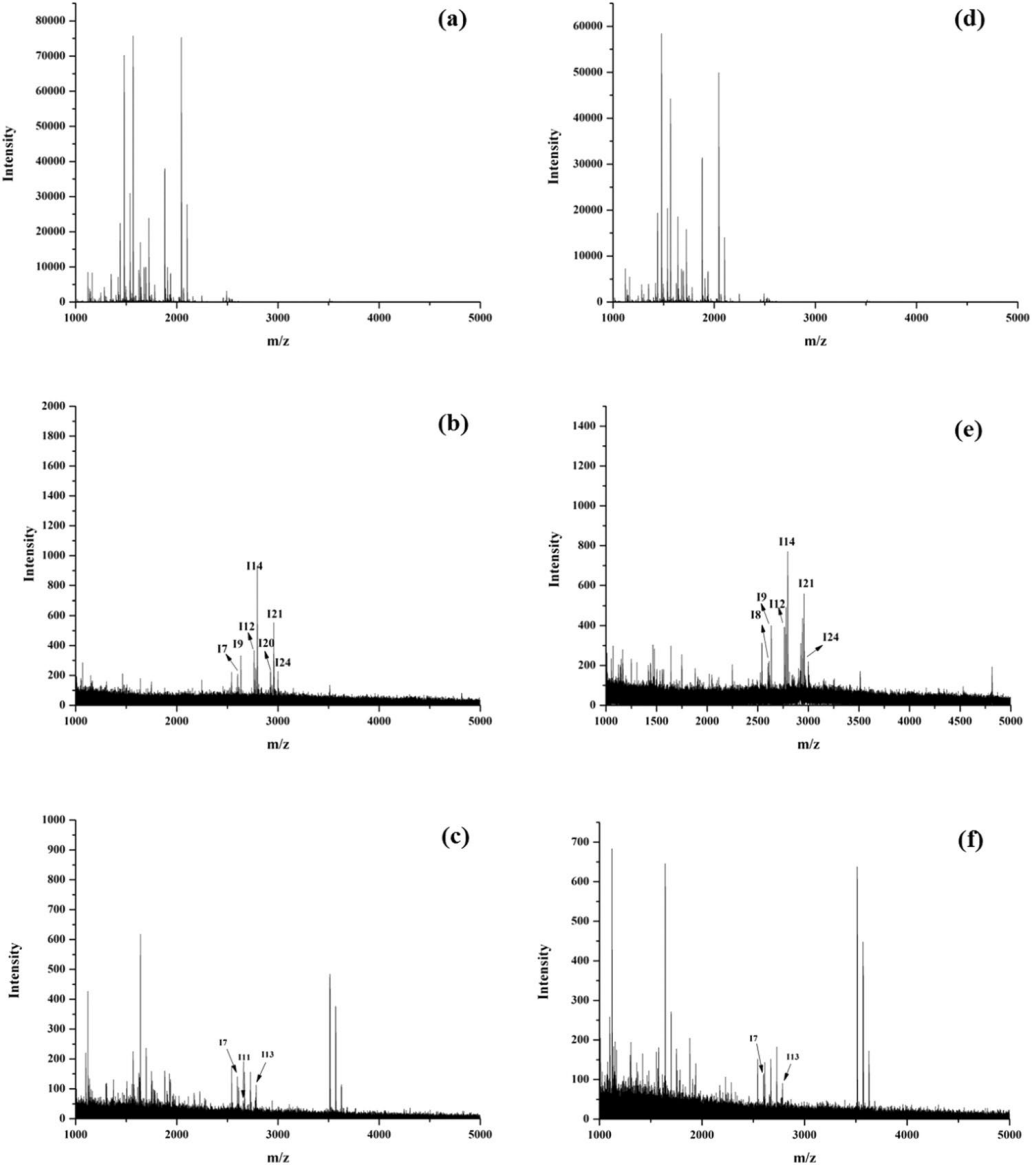
MALDI-TOF MS spectra of the mixture of human IgG and BSA tryptic digests (**a**) without enrichment, (**b**) after enrichment by Fe_3_O_4_@MPS@PMAC and (**c**) after enrichment by commercial HILIC (IgG: BSA = 1: 50, w/w, 1 mg/mL); (**d**) without enrichment, (**e**) after enrichment by Fe_3_O_4_@MPS@PMAC and (**f**) after enrichment by commercial HILIC (IgG: BSA = 1: 100, w/w, 1 mg/mL).

To evaluate the enrichment selectivity of Fe_3_O_4_@MPS@PMAC, the mixture of the tryptic digests of BSA and human IgG were incubated with the nanoparticles. As shown in Fig. 6a, when the mass ratio of human IgG:BSA was set to 1:50, no glycopeptides could be detected before enrichment as the desired signal was severely suppressed by the abundant presence of non-glycopeptides. After enrichment, 8 glycopeptides were observed, and the non-glycopeptide signals were nearly gone (Fig. 6b and Table S4). Next, the mass ratio of human IgG: BSA was further reduced to 1:100 and 10 glycopeptides could be detected after enrichment (Fig. 6e and Table S5). For comparison, the mixture of the tryptic digests of BSA and human IgG were also treated with commercial HILIC, only 3 glycopeptides together with many non-glycosylated peptides were detected when the mass ratio of human IgG:BSA was set to 1:50 (Fig. 6c). The number of identified glycopeptide decreased to 2 when the mass ratio of human IgG:BSA was further reduced to 1:100 (Fig. 6f). These results demonstrated the high glycopeptide enrichment selectivity of the synthesized Fe_3_O_4_@MPS@PMAC.

**Figure 7:**
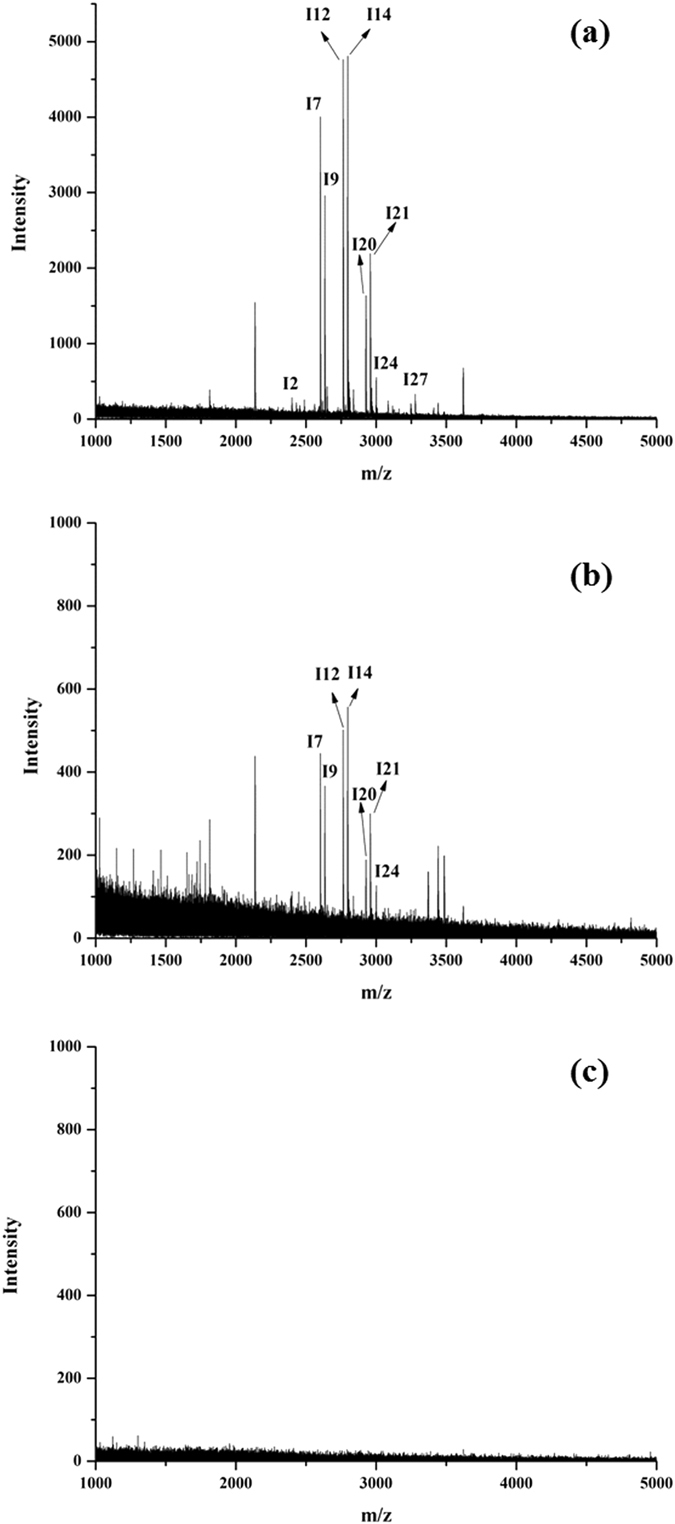
MALDI-TOF MS spectra of (**a**) 50 fmol, (**b**) 10 fmol human IgG tryptic digests after enrichment by Fe_3_O_4_@MPS@PMAC and (**c**) 10 fmol human IgG tryptic digests after enrichment by commercial HILIC.

Next, the tryptic digests of human IgG at different concentrations were used to evaluate the enrichment sensitivity of Fe_3_O_4_@MPS@PMAC. As shown in Fig. 7, 20 glycopeptide peaks could be identified after enrichment with 50 fmol human IgG (Fig. 7a and Table S6). The concentration was further decreased to 10 fmol, and 8 glycopeptide peaks could still be clearly identified (Fig. 7b and Table S7). However, the signal of the glycopeptide could not be detected after enrichment of 10 fmol tryptic digests of human IgG by commercial HILIC (Fig. 7c). The resulting detection sensitivity was higher than those with other HILIC materials such as silica-based click maltose (30 fmol)^46^ and L-Cys modified graphene oxide composites (25 fmol)^47^. The high detection sensitivity of the Fe_3_O_4_@MPS@PMAC made it possible for trace glycoprotein enrichment in proteomics sample analysis.

The binding capacity of the nanoparticles for glycopeptides was tested by adding different amounts of Fe_3_O_4_@MPS@PMAC (5–50 μg) to 3 μg of tryptic digests of human IgG. The enrichment procedure was conducted using the method described above and the eluent was analyzed by MALDI-TOF MS/MS. When 30 μg of Fe_3_O_4_@MPS@PMAC was added, the signal intensities of six selected peaks reached a maximum (Fig. S1). The binding capacity was calculated to be approximately 100 μg mg^−1^. The large binding capacity was due to significant modification of MAC and the strong hydrophilic interaction.

The recovery yield was also evaluated by the stable-isotope dimethyl labeling method^48^. Briefly, equal amounts of the human IgG (3 μg) tryptic digest were labelled with HCHO and DCDO. The heavy-isotope-tagged digest was enriched with Fe_3_O_4_@MPS@PMAC according to the procedure mentioned above. The eluent was collected and mixed with the light-isotope-tagged digest. The mixture was enriched by Fe_3_O_4_@MPS@PMAC again and the eluent was deglycosylated and analyzed by MALDI-TOF MS. Three parallel enrichment experiments were conducted, and the results are shown in Table 1 and Fig. S2. The recovery yield was above 80%, which was higher than those with other HILIC materials such as Hydrophilic GO/Fe_3_O_4_/Au/PEG nanocomposites (59.2%)^20^ and hydrophilic polysaccharide-functionalized macroporous adsorption resin (73%)^49^. The high recovery yield indicated that Fe_3_O_4_@MPS@PMAC is an ideal material for glycopeptide enrichment.

**Table 1:** Recovery yield of deglycosylated N-linked glycopeptides from human IgG digest.

**No**.	**m/z**	**Recovery ± S.D (%, n = 3)**
L1/H1	1186/1190	82.4 + 0.9
L2/H2	1218/1222	82.7 + 3.0

### N-glycopeptides enrichment from complex biological samples

Playing a central role in cellular signaling and a resource of disease biomarker, exosomes usually contain a variety of proteins including high-abundance proteins as well as a low abundance of post-translationally modified proteins. To test the performance of the Fe_3_O_4_@MPS@PMAC on the enrichment and identification of the glycoproteome in complex biological sample, HeLa cell exosomes (Fig. S3) were selected as real sample and commercial hydrophilic material was utilized as a control. In total, 1,274 N-glycopeptides from 536 glycoproteins were identified with the enrichment of Fe_3_O_4_@MPS@PMAC, which was the best result compared with those of the previously reported studies on exosome glycoproteins to the best of our knowledge. In comparison, 996 N-glycopeptides from 444 glycoproteins were identified by using the commercial material (Figs S4 and S5). This result demonstrated the superiority of Fe_3_O_4_@MPS@PMAC for glycoprotein analysis to that of the currently used enrichment material.

Gene Ontology (GO) enrichment analysis was performed on the identified glycoproteins from HeLa cell exosomes to examine the cellular components, molecular function, and biological processes in which they are involved. As shown in Fig. S6, for biological processes, these glycoproteins were mainly involved in “cell adhesion” and “extracellular matrix organization” as well as “cell migration”. For molecular function, most of the glycoproteins played an important role in protein binding and some of them related to receptor activity. With regard to the cellular component, the identified glycoproteins were mainly located at the “cell surface”, “extracellular exosome” and “membrane”. This result indicated that the identified glycoproteins mainly originate from the plasma membrane and are associated with cell adhesion and migration.

In summary, a novel nanomaterial, Fe_3_O_4_@MPS@PMAC, was successfully synthesized through the one-step reflux-precipitation polymerization. The hydrophilic ionic liquid was utilized for the synthesis of glycopeptides enrichment material for the first time. The nanoparticles showed high specificity, selectivity, sensitivity and recovery yield for glycopeptide enrichment with a standard glycoprotein compared with the commercial material. In the analysis with a real complex bio-sample, HeLa cell exosomes, Fe_3_O_4_@MPS@PMAC demonstrated its superiority and the feasibility for the efficiently enrichment of glycopeptides. This work provides a new avenue for glycoproteome research and broadens the research possibilities for the study of exosomes.

## Methods

### Chemicals and reagents

Iron(III) chloride hexahydrate (FeCl_3_·6H_2_O), chicken avidin (CA), human immunoglobulin G (IgG), bovine serum album (BSA), N,N’-methylenebisacrylamide (MBA), γ-methacryloxypropyltrimethoxysilane (MPS), tetraethyl orthosilicate (TEOS), triethylammonium bicarbonate buffer (TEAB), [2-(methacryloyloxy)ethyl]trimethylammonium chloride solution (MAC), 2,5-dihydroxybenzoic acid (DHB), ammonium bicarbonate (NH_4_HCO_3_), trifluoroacetic acid (TFA), formic acid (FA), formaldehyde (HCHO), formaldehyde-d2 (DCDO), TPCK-treated trypsin and peptide-N-glycosidase (PNGase F) were purchased from Sigma-Aldrich (St. Louis, MO, USA). Dithiothreitol (DTT) and iodoacetamide (IAA) were purchased from BioRad (Hercules, CA, USA). HPLC grade acetonitrile (ACN), ethanol and commercial HILIC (SeQuant, ZIC-HILIC, 3.5 μm) were purchased from Merck (Darmstadt, Germany). Sodium acetate (NaAc), ethylene glycol (EG) and ammonium solution (37%) were purchased from Sinopharm Chemical Reagent Company (Shanghai, China). Ultrapure water was prepared from a Millipore purification system (Billerica, MA, USA). All other chemical agents were obtained from Shanghai Chemical Reagent.

### Synthesis of Fe_3_O_4_@MPS@PMAC nanoparticles

The Fe_3_O_4_ nanoparticles were prepared by a solvothermal reaction and coated with a layer of silica for the modification of γ-MPS. In brief, 160 mg FeCl_3_·6H_2_O and 30 mg sodium citrate were dispersed in 30 mL of ethylene glycol, followed by sonication for 5 min. Then, 700 mg NaAc was added into the mixture and stirred for 30 min. The solution was then transferred to a Teflon stainless steel autoclave set to 200 °C for 6 h. The resulting nanoparticles were collected with a magnet and washed sequentially with ethanol and water three times. The obtained Fe_3_O_4_ nanoparticles were dispersed in a solution of 25 mL ethanol, 4 mL H_2_O and 0.7 mL aqueous ammonia under sonication. Then, 1 mL TEOS was added dropwise into the solution followed by a 6 h reaction to form the silica layer. The composite was separated by a magnet and washed sequentially with water and isopropanol three times. The obtained nanoparticles were dispersed in the mixture of 40 mL of ethanol, 10 mL of water, 1.5 mL of NH_3_·H_2_O and 1 mL of γ-MPS. The mixture was stirred for 24 h at 60 °C. The obtained product was separated by a magnet, washed with ethanol, and dried at 50 °C overnight.

The PMAC layer was coated by a one-step reflux-precipitation polymerization (RPP) of MAC, using MBA as cross-linker and AIBN as an initiator. Specifically, 60 mg of Fe_3_O_4_@MPS was dispersed in 40 mL of ACN in a dried 100 mL round-bottom flask. Then 200 μL MAC, 200 mg MBA and 5 mg AIBN were added into the flask. After 30 min of ultrasonic treatment, the flask was transferred to a hot oil bath. The mixture was heated to 95 °C within 30 min and temperature was maintained for 1.5 h. The Fe_3_O_4_@MPS@PMAC nanoparticles were collected with a magnet and washed with ethanol and water three times. Finally, the nanoparticles were dried at 50 °C overnight.

### Material characterization

Transmission electron microscopy (TEM) images of the Fe_3_O_4_@MPS@PMAC nanoparticles were obtained by an FEI Tecnai G2 20 transmission electron microscope operating at 200 kV. Field-emission scanning electron (FE-SEM) images were recorded on a Hitachi S-4800 cold field-emission scanning electron microscope (Hitachi, Tokyo, Japan) equipped with an energy-dispersive X-ray spectrometer (EDX). Fourier transform infrared spectroscopy (FT-IR) characterization was performed using a Fourier spectrophotometer with KBr pellets (Nicolet, Wisconsin, USA). Thermogravimetric analysis (TGA) was carried out under nitrogen flow at a heating rate of 10 °C/min from 25 °C to 700 °C on a Pyris 1 TGA instrument (Perkin Elmer, Massachusetts, USA). All nanoparticles were dried at 60 °C prior to each TGA measurement to remove the solution attached to the surface. The saturation magnetization curves were measured at room temperature with a Physical Property Measurement System 9 T (Quantum Design, San Diego, USA).

### Digestion of standard glycoproteins

Standard glycoprotein (Human IgG or CA) was dissolved in 1 mL of 50 mM NH_4_HCO_3_ (pH 8.3) to a final concentration of 1 mg/mL. After denaturation at 90 °C for 10 min, the proteins were reduced in 10 mM DTT for 4 h at 37 °C and alkylated in the dark in 20 mM IAA for 1 h. Subsequently, trypsin was added to the solution (enzyme:protein 1:50, w/w) and incubated at 37 °C overnight. The tryptic digest was desalted, lyophilized, and stored at −20 °C for further use.

HeLa exosomes were isolated in the supernatant, which was obtained by differential ultracentrifugation of cultured cells^50^. Bovine serum used for culturing these cells was subjected to ultracentrifugation to remove exosomes prior to use. The cell culture medium was collected after each passage for exosome isolation. The method of exosome isolation is shown in Fig. S7. The morphology of extracted exosomes was investigated by transmission electron microscopy (TEM). After isolation, the exosomes were washed with PBS and resuspended in an ice-cold lysis buffer consisting of 4% SDS (w/v), a protease inhibitor mixture (1 mM PMSF, 0.2 mM Na_3_VO_4_, 1 mM NaF), and 100 mM Tris-HCl at a pH of 7.4. The mixture was sonicated at 100 W for 30 min. Finally, the sample was centrifuged at 14000 × g for 10 min at 4 °C, and the supernatant was collected for protein digestion. The extracted exosome proteins were denatured at 95 °C for 5 min. After reduction by 10 mM DTT at 37 °C for 4 h and alkylation by 20 mM IAA at room temperature in the dark for 1 h, free trypsin was added into the protein solution at a ratio of 1:50. This solution was incubated at 37 °C overnight. The digests were desalted prior to glycopeptide enrichment.

### Enrichment of N-link glycopeptides with Fe_3_O_4_@MPS@PMAC nanoparticles

The enrichment process was shown in Fig. S8. First, 20 μg of Fe_3_O_4_@MPS@PMAC nanoparticles or commercial HILIC were washed and dispersed in 50 μL of loading buffer (ACN/H_2_O/TFA, 88:11.9:0.1, v/v/v). Then, 1 μg digested glycoprotein (human IgG or CA) was added to the mixture, which was shaken for 30 min at room temperature. After incubation, the glycopeptide-loaded Fe_3_O_4_@MPS@PMAC was separated with a magnet and washed three times with loading buffer (100 μL) to remove non-specific adsorbed peptides (centrifugation for commercial HILIC). Then, the captured glycopeptides were eluted by washing twice with 20 μL of elution buffer (ACN/H_2_O/TFA, 30:69.9:0.1, v/v/v). The ﻿﻿obtained glycopeptides were subjected to MALDI-TOF-MS for further analysis.

For HeLa exosome proteins, 1 mg of Fe_3_O_4_@MPS@PMAC nanoparticles or commercial HILIC were incubated with 50 μg of the HeLa exosome protein tryptic digests in a 200 μL loading buffer (ACN/H_2_O/TFA, 88:11:1, v/v/v). After vigorous shaking for 1 h, the nanoparticles were subsequently washed three times with 200 μL loading buffer. Then, the captured glycopeptides were eluted by washing twice with 100 μL of eluting buffer (ACN/H_2_O/TFA, 30:69.9:0.1, v/v/v). The collected eluate was combined, lyophilized, and deglycosylated for LC-MS/MS analysis.

### Deglycosylation of N-linked glycopeptides by PNGase F with ^18^O labeling

The captured exosomes glycopeptides were lyophilized and dissolved in 50 μL 50 mM NH_4_HCO_3_ (pH = 8.3, H_2_^18^O) solution. Then, 100 U of PNGase F were added to the solution and the mixture was incubated at 37 °C overnight. The deglycosylated peptides were further desalted, lyophilized and dissolved in 10 μL 0.1% FA for MALDI-TOF-MS or LC-MS analysis.

### Mass spectrometry analysis

Standard glycopeptide analyses were performed on a Bruker Ultraflex III MALDI-TOF/TOF MS (Bruker, Daltonics, Germany). One microliter of sample was dropped on the MALDI plate and dried at room temperature. Then, 1 μL of DHB matrix solution (20 mg/mL, ACN/H_2_O/TFA, 60:39.9:0.1, v/v/v) was spotted on the MALDI plate and air dried. For HeLa exosome proteins, the LC-MS/MS analyses was carried out using an easy nLC-1000 system coupled with a Fusion Lumos mass spectrometer (Thermo Fisher Scientific, USA) using an ESI nanospray source. Mobile phase A was composed of 0.1% FA in water, and 0.1% FA in ACN was prepared as mobile phase B. The total flow rate was 600 nL/min, and the gradient was performed as follows: 6% to 9% buffer B for 8 min; 9% to 14% buffer B for 16 min; 14% to 30% buffer B for 36 min; 30% to 40% buffer B for 15 min; and 40% to 95% buffer B for 3 min. After eluting with 95% buffer for 7 min, the separation system was equilibrated with 6% buffer B for 5 min. The spray voltage was operated at 2.3 kV. The MS/MS spectra were acquired in data-dependent acquisition mode, and the full mass scan was acquired for m/z from 300 to 1,400 with a resolution of 120,000.

### Database searching

The acquired LC-MS/MS data files were analyzed by Maxquant (version 1.5.2.8), with the spectra searched against the UniProt Human database (updated on July ^21th^, 2015). The search criteria were set as follows: fixed modification of cysteine residues (+57.0215 Da), variable modification of methionine oxidation, deglycosylated asparagine ^18^O labeling (+2.9890 Da), asparagine and glutamine deamination (+0.9840 Da). The mass tolerances were 20 ppm for initial precursor ions and 0.5 Da for fragment ions. Two missed cleavages in tryptic digests were allowed. Filtering for the peptide identification was set at a 1% false discovery rate (FDR). Only identified peptides with the sequence of N-!P-S/C/T were considered as N-linked glycopeptides.

## Electronic supplementary material

Supplementary Information

Supplementary Dataset
