# Supplementary material for: Polymeric hydrophilic ionic liquids used to modify magnetic nanoparticles for the highly selective enrichment of N-linked glycopeptides
Source: Sci Rep. 2017 Aug 1;7:6984. doi: 10.1038/s41598-017-07516-x (PMC5539331; doi:10.1038/s41598-017-07516-x)
Supplement: Supplementary file 1 — Supplementary Information [file 41598_2017_7516_MOESM1_ESM.docx]

**Polymeric hydrophilic ionic liquids used to modify magnetic nanoparticles for the highly selective enrichment of N-linked glycopeptides**

Fenglong Jiao, ^1,2^ Fangyuan Gao, ^2^ Heping Wang, ^2^ Yulin Deng, ^1^ Yangjun Zhang, ^2,*^ Xiaohong Qian, ^2,*^ & Yukui Zhang, ^3^

^1^School of Life Science and Technology, Beijing Institute of Technology, Beijing 100081, China, ^2^State Key Laboratory of Proteomics, National Center for Protein Science, Beijing Institute of Radiation Medicine, Beijing 102200, China, ^3^National Chromatographic Research and Analysis Center, Dalian Institute of Chemical Physics, Chinese Academy of Sciences, Dalian 116011, China

* Correspondence to:

Yangjun Zhang

State Key Laboratory of Proteomics

National Center for Protein Science Beijing,

Beijing Institute of Radiation Medicine,

Beijing 102200, China

Email: [13683167093@163.com](mailto:13683167093@163.com)

Tel/Fax: +86-10-61777110

Xiaohong Qian

State Key Laboratory of Proteomics

National Center for Protein Science Beijing,

Beijing Institute of Radiation Medicine,

Beijing 102200, China

Email: [13911734119@163.com](mailto:13911734119@163.com)

Tel/Fax: +86-10-61777109

**Table S1.** Energy dispersive X-ray (EDX) analysis of Fe_3_O_4,_ Fe_3_O_4_@MPS and Fe_3_O_4_@MPS@PMAC

| **Element** | **Fe_3_O_4_** | | **Fe_3_O_4_@MPS** | | **Fe_3_O_4_@MPS@PMAC** | |
| --- | --- | --- | --- | --- | --- | --- |
|  | **Wt%** | **At%** | **Wt%** | **At%** | **Wt%** | **At%** |
| **C** | **2.91** | **7.56** | **4.76** | **10.89** | **27.05** | **43.60** |
| **N** | **1.20** | **2.68** | **1.32** | **2.58** | **4.34** | **6.00** |
| **O** | **25.74** | **50.30** | **30.35** | **52.11** | **29.17** | **35.30** |
| **Si** | **0.35** | **0.39** | **6.48** | **6.34** | **2.62** | **1.80** |
| **Cl** | **-** | **-** | **-** | **-** | **2.69** | **1.47** |
| **Fe** | **69.80** | **39.07** | **57.09** | **28.08** | **34.14** | **11.84** |

**Table S2.** Molecular masses and proposed oligosaccharide composition of glycopeptides enriched from human IgG digest. N# denotes the N-linked glycosylation site.

| **Peak number** | **Observed m/z** | **Glycan composite** | **Amino acid sequence** |
| --- | --- | --- | --- |
| **1** | **2286** | **[Hex]3[HexNAc]3** | **EEQYN#STYR** |
| **2** | **2400** | **[Hex]3[HexNAc]3[Fuc]1** | **EEQFN#STFR** |
| **3** | **2432** | **[Hex]3[HexNAc]3[Fuc]1** | **EEQYN#STYR** |
| **4** | **2488** | **[Hex]3[HexNAc]4** | **EEQYN#STYR** |
| **5** | **2562** | **[Hex]4[HexNAc]3[Fuc]1** | **EEQFN#STFR** |
| **6** | **2594** | **[Hex]4[HexNAc]3[Fuc]1** | **EEQYN#STYR** |
| **7** | **2603** | **[Hex]3[HexNAc]4[Fuc]1** | **EEQFN#STFR** |
| **8** | **2618** | **[Hex]4[HexNAc]4** | **EEQFN#STFR** |
| **9** | **2635** | **[Hex]3[HexNAc]4[Fuc]1** | **EEQYN#STYR** |
| **10** | **2650** | **[Hex]4[HexNAc]4** | **EEQYN#STYR** |
| **11** | **2658** | **[Hex]3[HexNAc]5** | **EEQFN#STYR** |
| **12** | **2764** | **[Hex]4[HexNAc]4[Fuc]1** | **EEQFN#STFR** |
| **13** | **2780** | **[Hex]5[HexNAc]4** | **EEQFN#STFR** |
| **14** | **2797** | **[Hex]4[HexNAc]4[Fuc]1** | **EEQYN#STYR** |
| **15** | **2806** | **[Hex]3[HexNAc]5[Fuc]1** | **EEQFN#STYR** |
| **16** | **2812** | **[Hex]5[HexNAc]4** | **EEQYN#STFR** |
| **17** | **2821** | **[Hex]4[HexNAc]5** | **EEQFN#STFR** |
| **18** | **2838** | **[Hex]3[HexNAc]5[Fuc]1** | **EEQYN#STYR** |
| **19** | **2853** | **[Hex]4[HexNAc]5** | **EEQYN#STYR** |
| **20** | **2926** | **[Hex]5[HexNAc]4[Fuc]1** | **EEQFN#STFR** |
| **21** | **2958** | **[Hex]5[HexNAc]4[Fuc]1** | **EEQYN#STYR** |
| **22** | **2968** | **[Hex]4[Hex7NAc]5[Fuc]1** | **EEQFN#STFR** |
| **23** | **2983** | **[Hex]5[HexNAc]5** | **EEQFN#STFR** |
| **24** | **3000** | **[Hex]4[HexNAc]5[Fuc]1** | **EEQYN#STYR** |
| **25** | **3129** | **[Hex]5[HexNAc]5[Fuc]1** | **EEQFN#STFR** |
| **26** | **3161** | **[Hex]5[HexNAc]5[Fuc]1** | **EEQYN#STYR** |
| **27** | **3250** | **[Hex]5[HexNAc]4[Fuc]1[NeuAc]1** | **EEQYN#STYR** |

**HexNAc=N-acetylglucosamine, Hex=mannose, Fuc=fuctose, NeuAc=sialic.**

**Table S3.** Molecular masses and proposed oligosaccharide composition of glycopeptides enriched from chicken avidin digest. N# denotes the N-linked glycosylation site.

| **Peak number** | **Observed m/z** | **Glycan composite** | **Amino acid sequence** |
| --- | --- | --- | --- |
| **1** | **2039** | **[HexNAc]1** | **WTNDLGSN#MTIGAVNSR** |
| **2** | **2728** | **[Hex]3[HexNAc]2** | **WTNDLGSN#MTIGAVNSR** |
| **3** | **2890** | **[Hex]4[HexNAc]2** | **WTNDLGSN#MTIGAVNSR** |
| **4** | **2931** | **[Hex]2[HexNAc]3** | **WTNDLGSN#MTIGAVNSR** |
| **5** | **3052** | **[Hex]5[HexNAc]2** | **WTNDLGSN#MTIGAVNSR** |
| **6** | **3093** | **[Hex]3[HexNAc]3** | **WTNDLGSN#MTIGAVNSR** |
| **7** | **3135** | **[Hex]4[HexNAc]3** | **WTNDLGSN#MTIGAVNSR** |
| **8** | **3214** | **[Hex]6[HexNAc]2** | **WTNDLGSN#MTIGAVNSR** |
| **9** | **3255** | **[Hex]5[HexNAc]3** | **WTNDLGSN#MTIGAVNSR** |
| **10** | **3296** | **[Hex]4[HexNAc]4** | **WTNDLGSN#MTIGAVNSR** |
| **11** | **3376** | **[Hex]7[HexNAc]2** | **WTNDLGSN#MTIGAVNSR** |
| **12** | **3417** | **[Hex]6[HexNAc]3** | **WTNDLGSN#MTIGAVNSR** |
| **13** | **3458** | **[Hex]5[HexNAc]4** | **WTNDLGSN#MTIGAVNSR** |
| **14** | **3620** | **[Hex]6[HexNAc]4** | **WTNDLGSN#MTIGAVNSR** |

**HexNAc=N-acetylglucosamine, Hex=mannose.**

**Table S4.** Molecular masses and proposed oligosaccharide composition of glycopeptides enriched from mixture of BSA and human IgG digest (50:1). N# denotes the N-linked glycosylation site.

| **Peak number** | **Observed m/z** | **Glycan composite** | **Amino acid sequence** |
| --- | --- | --- | --- |
| **1** | **2603** | **[Hex]3[HexNAc]4[Fuc]1** | **EEQFN#STFR** |
| **2** | **2635** | **[Hex]3[HexNAc]4[Fuc]1** | **EEQYN#STYR** |
| **3** | **2764** | **[Hex]4[HexNAc]4[Fuc]1** | **EEQFN#STFR** |
| **4** | **2780** | **[Hex]4[HexNAc]4[Fuc]1** | **EEQYN#STYR** |
| **5** | **2797** | **[Hex]4[HexNAc]4[Fuc]1** | **EEQYN#STYR** |
| **6** | **2926** | **[Hex]5[HexNAc]4[Fuc]1** | **EEQFN#STFR** |
| **7** | **2958** | **[Hex]5[HexNAc]4[Fuc]1** | **EEQYN#STYR** |
| **8** | **3002** | **[Hex]4[HexNAc]5[Fuc]1** | **EEQYN#STYR** |

**HexNAc=N-acetylglucosamine, Hex=mannose, Fuc=fuctose, NeuAc=sialic.**

**Table S5.** Molecular masses and proposed oligosaccharide composition of glycopeptides enriched from mixture of BSA and human IgG digest (100:1). N# denotes the N-linked glycosylation site.

| **Peak number** | **Observed m/z** | **Glycan composite** | **Amino acid sequence** |
| --- | --- | --- | --- |
| **1** | **2603** | **[Hex]3[HexNAc]4[Fuc]1** | **EEQFN#STFR** |
| **2** | **2618** | **[Hex]4[HexNAc]4** | **EEQFN#STFR** |
| **3** | **2635** | **[Hex]3[HexNAc]4[Fuc]1** | **EEQYN#STYR** |
| **4** | **2764** | **[Hex]4[HexNAc]4[Fuc]1** | **EEQFN#STFR** |
| **5** | **2780** | **[Hex]5[HexNAc]4** | **EEQFN#STFR** |
| **6** | **2797** | **[Hex]4[HexNAc]4[Fuc]1** | **EEQYN#STYR** |
| **7** | **2812** | **[Hex]5[HexNAc]4** | **EEQYN#STFR** |
| **8** | **2925** | **[Hex]5[HexNAc]4[Fuc]1** | **EEQFN#STFR** |
| **9** | **2956** | **[Hex]5[HexNAc]4[Fuc]1** | **EEQYN#STYR** |
| **10** | **3000** | **[Hex]4[HexNAc]5[Fuc]1** | **EEQYN#STYR** |

**HexNAc=N-acetylglucosamine, Hex=mannose, Fuc=fuctose, NeuAc=sialic.**

**Table S6.** Molecular masses and proposed oligosaccharide composition of glycopeptides enriched from 50 fmol human IgG digest. N# denotes the N-linked glycosylation site.

| **Peak number** | **Observed m/z** | **Glycan composite** | **Amino acid sequence** |
| --- | --- | --- | --- |
| **1** | **2400** | **[Hex]3[HexNAc]3[Fuc]1** | **EEQFN#STFR** |
| **2** | **2432** | **[Hex]3[HexNAc]3[Fuc]1** | **EEQYN#STYR** |
| **3** | **2488** | **[Hex]3[HexNAc]4** | **EEQYN#STYR** |
| **4** | **2562** | **[Hex]4[HexNAc]3[Fuc]1** | **EEQFN#STFR** |
| **5** | **2594** | **[Hex]4[HexNAc]3[Fuc]1** | **EEQYN#STYR** |
| **6** | **2603** | **[Hex]3[HexNAc]4[Fuc]1** | **EEQFN#STFR** |
| **7** | **2618** | **[Hex]4[HexNAc]4** | **EEQFN#STFR** |
| **8** | **2635** | **[Hex]3[HexNAc]4[Fuc]1** | **EEQYN#STYR** |
| **9** | **2650** | **[Hex]4[HexNAc]4** | **EEQYN#STYR** |
| **10** | **2764** | **[Hex]4[HexNAc]4[Fuc]1** | **EEQFN#STFR** |
| **11** | **2780** | **[Hex]5[HexNAc]4** | **EEQFN#STFR** |
| **12** | **2797** | **[Hex]4[HexNAc]4[Fuc]1** | **EEQYN#STYR** |
| **13** | **2806** | **[Hex]3[HexNAc]5[Fuc]1** | **EEQFN#STYR** |
| **14** | **2812** | **[Hex]5[HexNAc]4** | **EEQYN#STFR** |
| **15** | **2838** | **[Hex]3[HexNAc]5[Fuc]1** | **EEQYN#STYR** |
| **16** | **2926** | **[Hex]5[HexNAc]4[Fuc]1** | **EEQFN#STFR** |
| **17** | **2958** | **[Hex]5[HexNAc]4[Fuc]1** | **EEQYN#STYR** |
| **18** | **2968** | **[Hex]4[Hex7NAc]5[Fuc]1** | **EEQFN#STFR** |
| **19** | **3000** | **[Hex]4[HexNAc]5[Fuc]1** | **EEQYN#STYR** |
| **20** | **3250** | **[Hex]5[HexNAc]4[Fuc]1[NeuAc]1** | **EEQYN#STYR** |

**HexNAc=N-acetylglucosamine, Hex=mannose, Fuc=fuctose, NeuAc=sialic.**

**Table S7.** Molecular masses and proposed oligosaccharide composition of glycopeptides enriched from 10 fmol human IgG digest. N# denotes the N-linked glycosylation site.

| **Peak number** | **Observed m/z** | **Glycan composite** | **Amino acid sequence** |
| --- | --- | --- | --- |
| **1** | **2603** | **[Hex]3[HexNAc]4[Fuc]1** | **EEQFN#STFR** |
| **2** | **2635** | **[Hex]3[HexNAc]4[Fuc]1** | **EEQYN#STYR** |
| **3** | **2764** | **[Hex]4[HexNAc]4[Fuc]1** | **EEQFN#STFR** |
| **4** | **2797** | **[Hex]4[HexNAc]4[Fuc]1** | **EEQYN#STYR** |
| **5** | **2838** | **[Hex]3[HexNAc]5[Fuc]1** | **EEQYN#STYR** |
| **6** | **2926** | **[Hex]5[HexNAc]4[Fuc]1** | **EEQFN#STFR** |
| **7** | **2958** | **[Hex]5[HexNAc]4[Fuc]1** | **EEQYN#STYR** |
| **8** | **3000** | **[Hex]4[HexNAc]5[Fuc]1** | **EEQYN#STYR** |

**HexNAc=N-acetylglucosamine, Hex=mannose, Fuc=fuctose, NeuAc=sialic.**


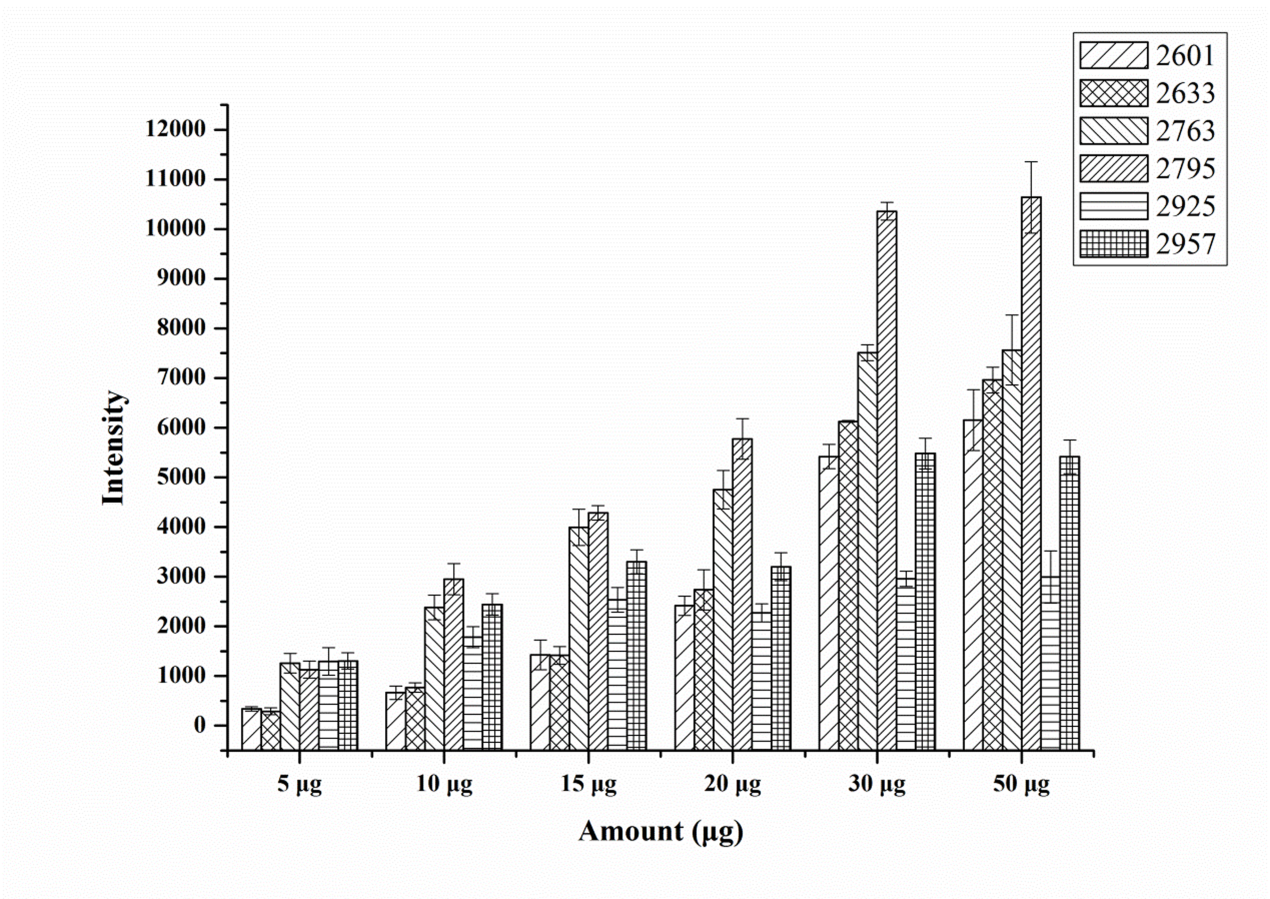


**Figure S1.** Signal intensity of six selected glycopeptides from human IgG tryptic digests after enrichment with different amounts of Fe_3_O_4_@MPS@PMAC


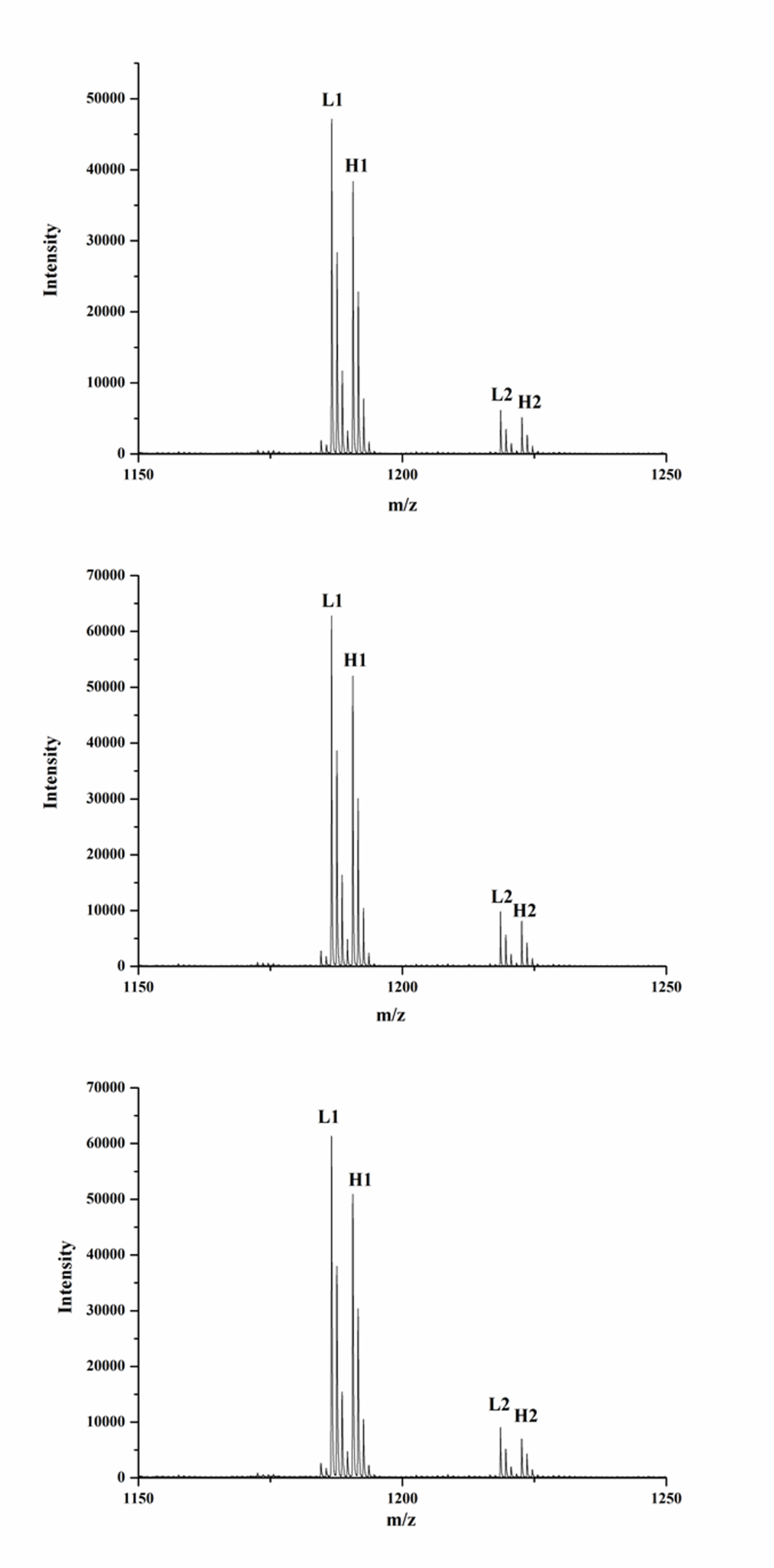


**Figure S2.** MALDI-TOF MS spectra of three paralleled experiments for evaluation of the recovery yield. L and H indicate the light and heavy tagged N-linked glycopeptide, respectively.


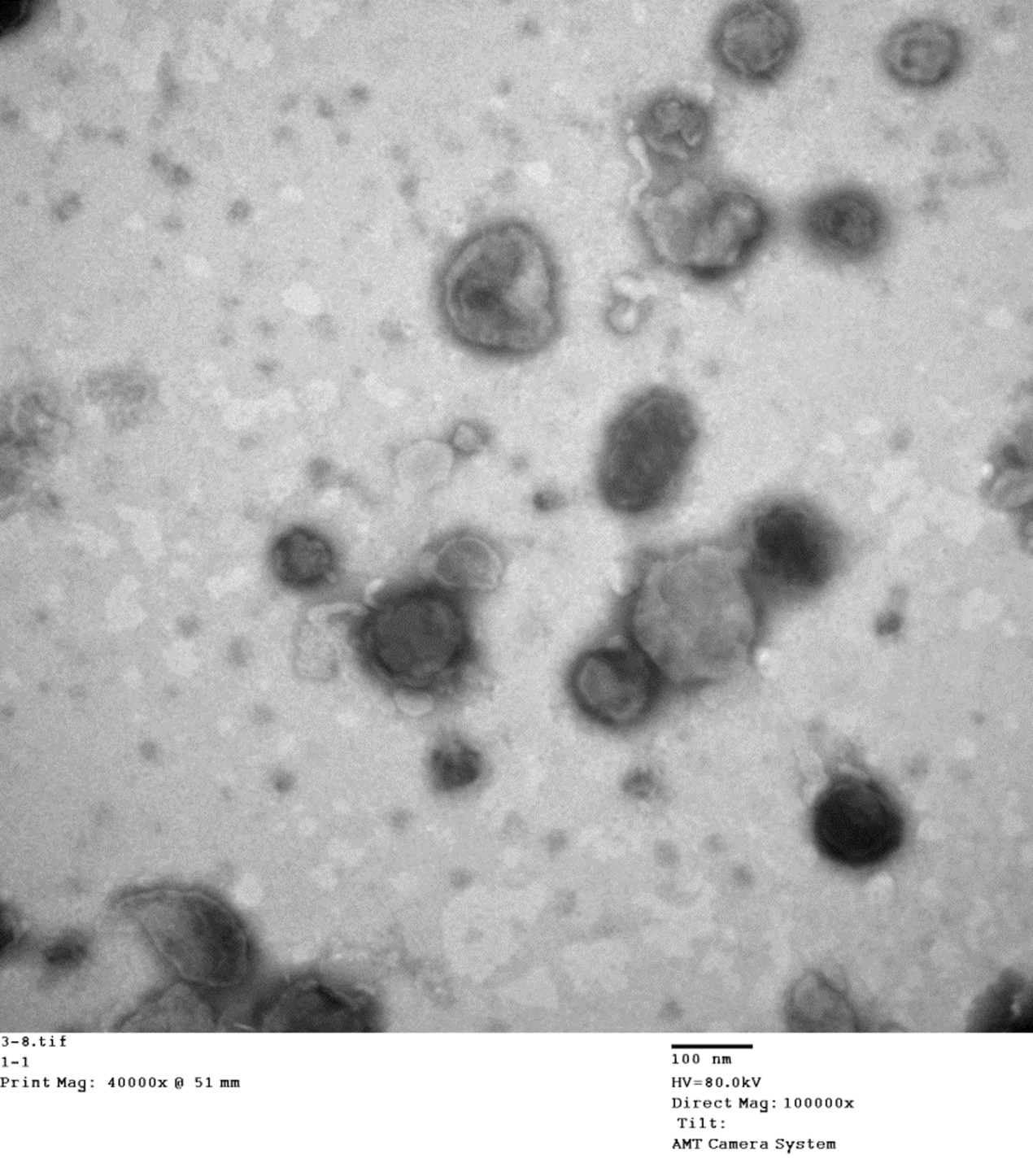


**Figure S3.** TEM image of exosomes extracted from HeLa cells


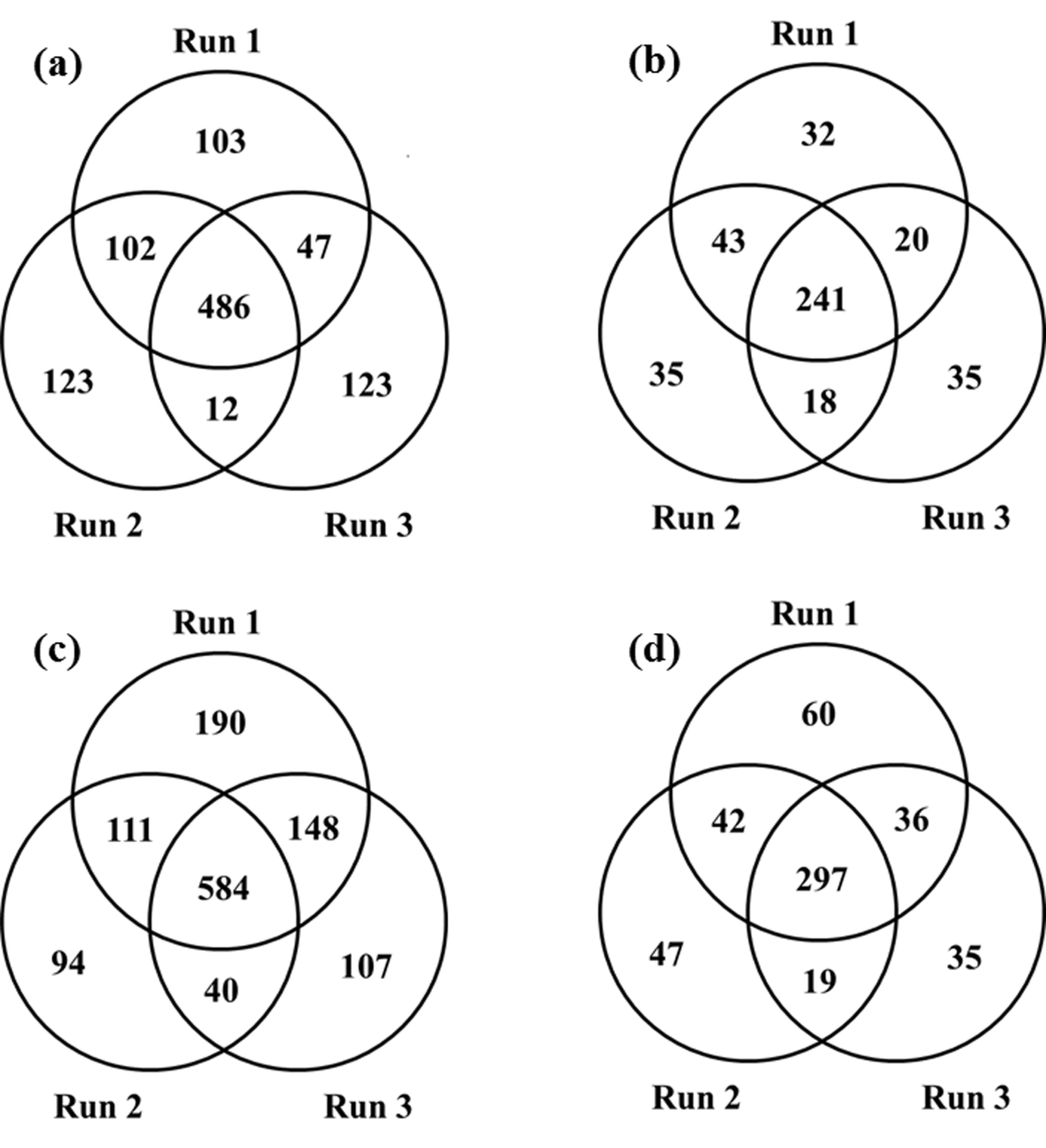


**Figure S4.** Data of three independent LC/MS runs of N-linked glycopeptides (left) and corresponded N-linked glycoproteins (right) enriched by (a) and (b) commercial hydrophilic materials, (c) and (d) Fe_3_O_4_@MPS@PMAC from tryptic digest of HeLa exosomes.


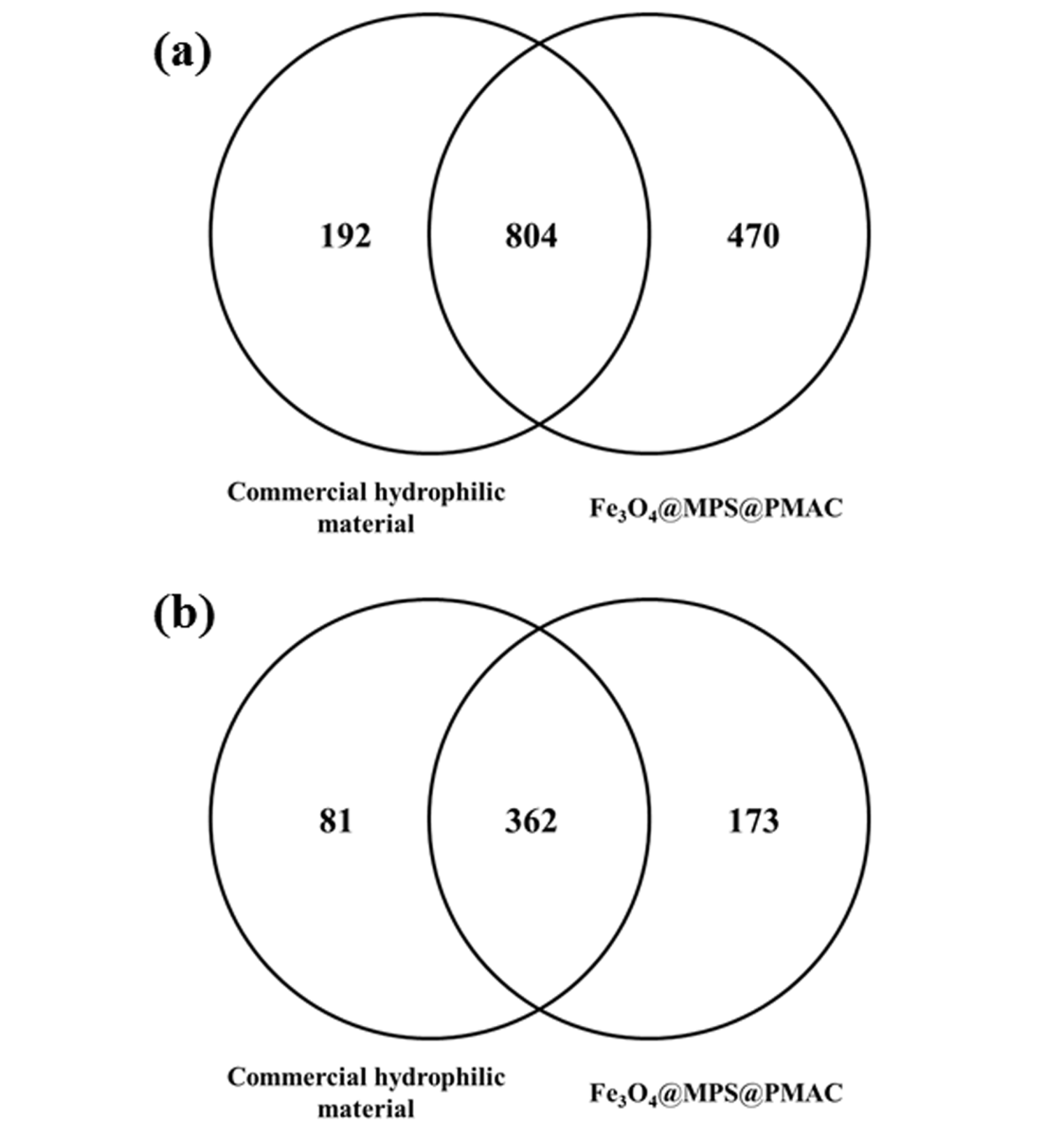


**Figure S5.** Overlap of identified N-linked glycopeptides (a) and corresponded N-linked glycoproteins (b) from tryptic digests of HeLa exosomes after enrichment by commercial hydrophilic materials and Fe_3_O_4_@MPS@PMAC


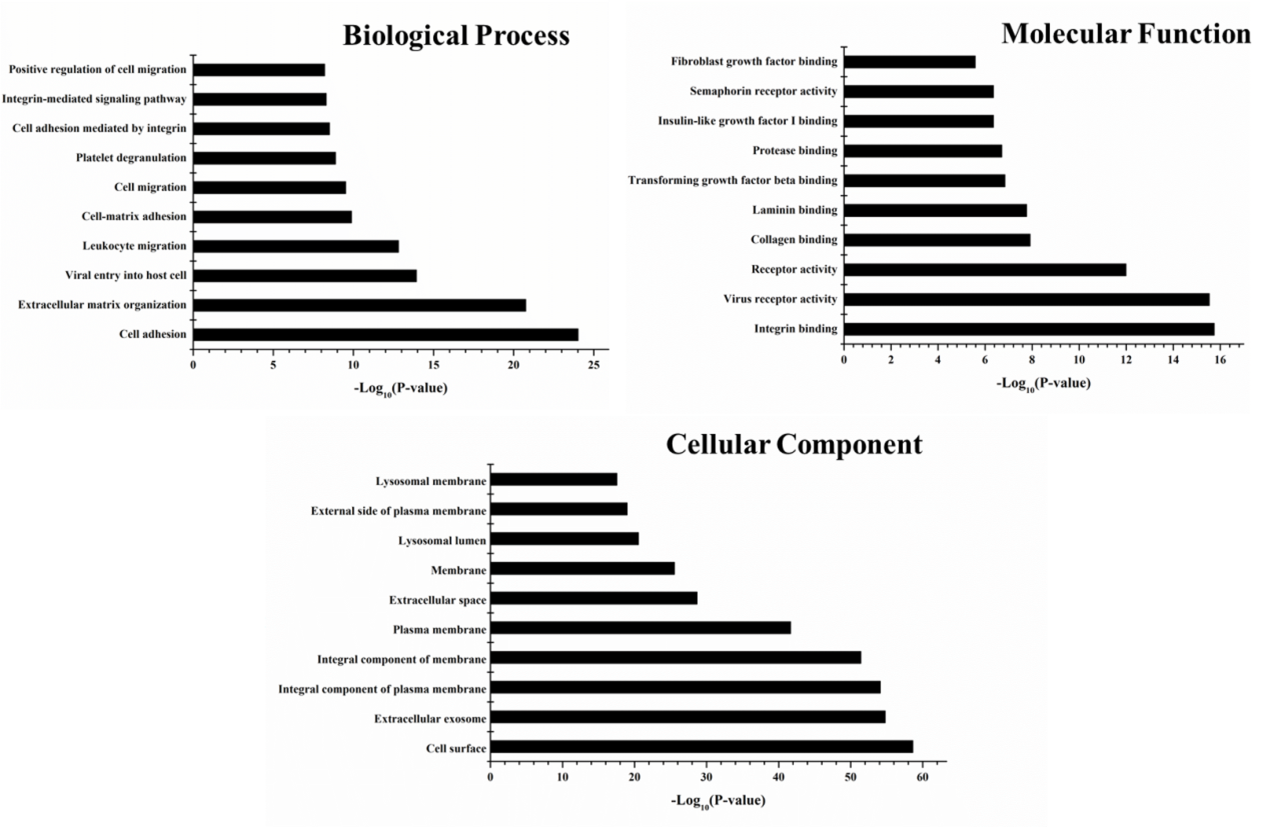


**Figure S6.** GO enrichment analysis of HeLa exosome glycoproteins. The top 10 significantly enriched GO categories under biological process, molecular function and cellular component were indicated.


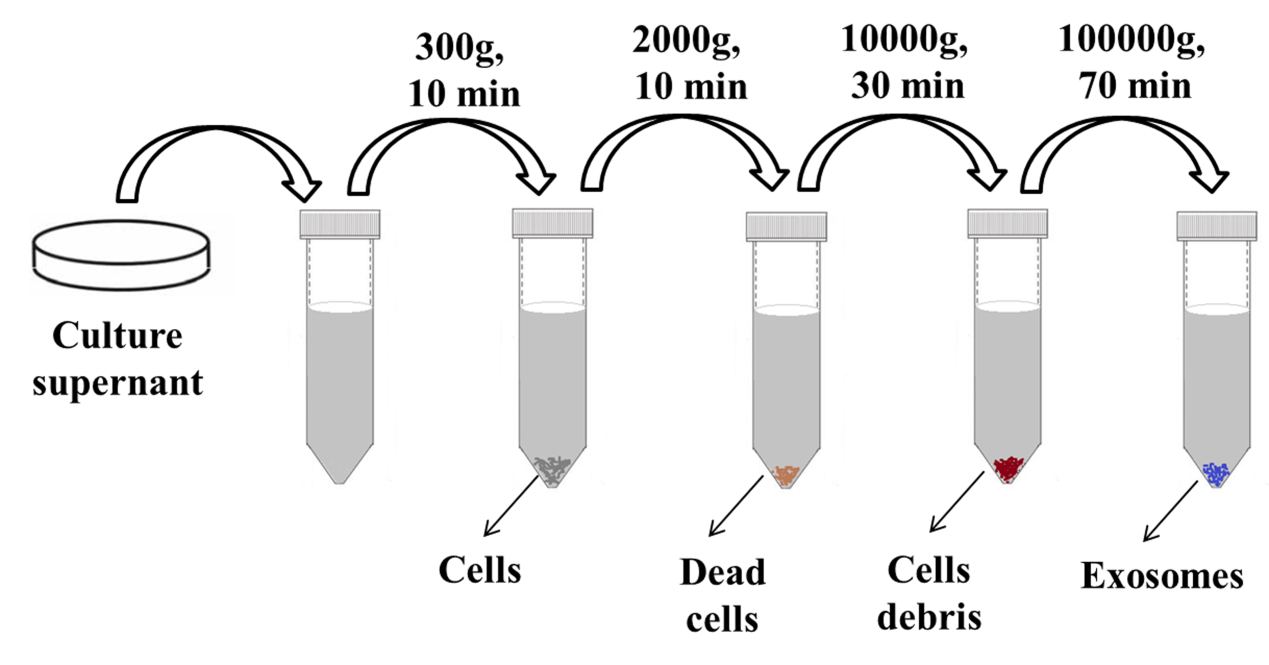


**Figure S7.** Procedure for exosome purification based on differential ultracentrifugation


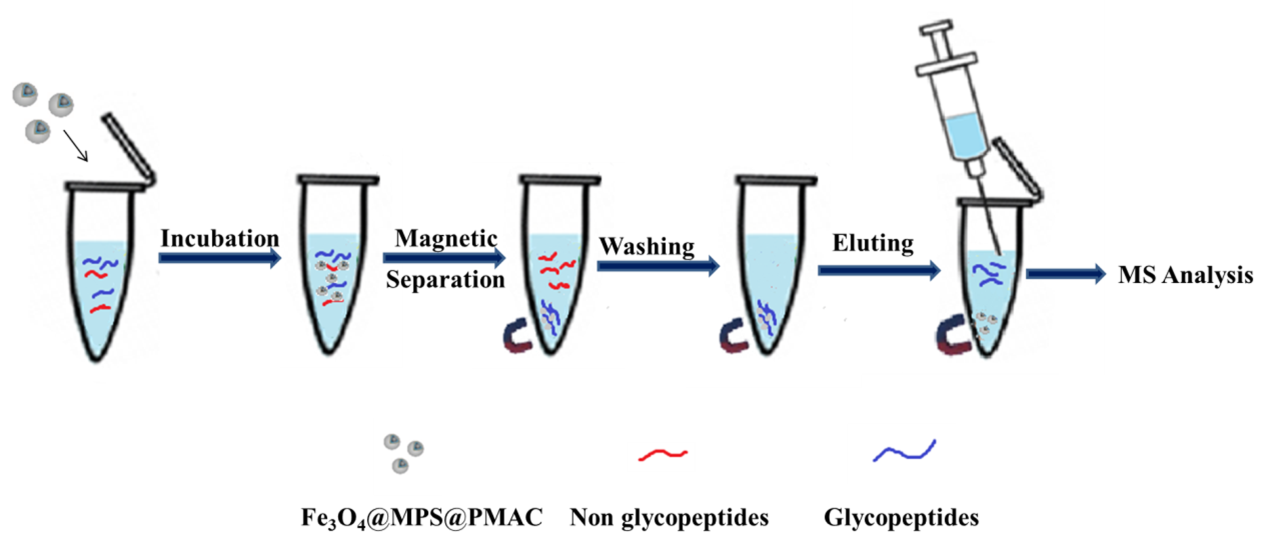


**Figure S8.** Procedure for glycopeptides enrichment by Fe_3_O_4_@MPS@PMAC


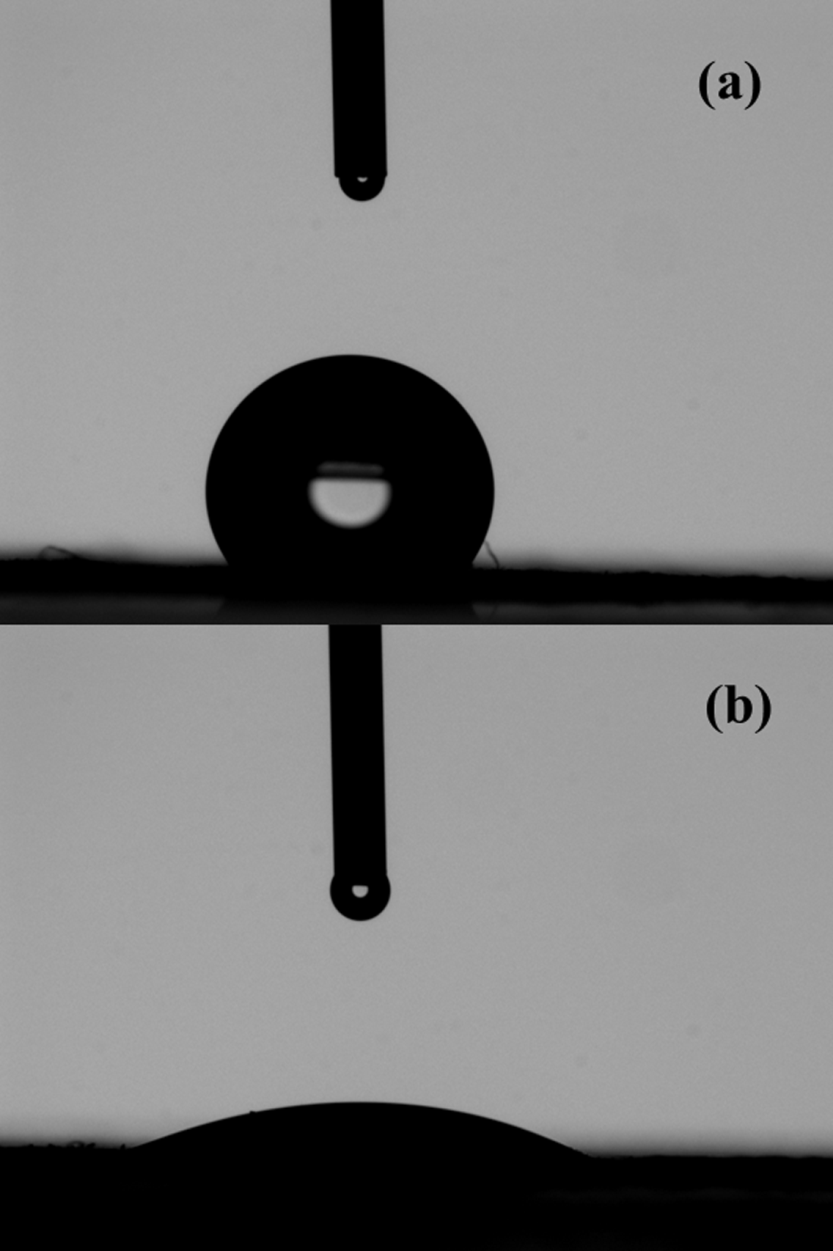


**Figure S9.** Contact angles of (a) Fe_3_O_4_@MPS and (b) Fe_3_O_4_@MPS@PMAC, detected when the drop just contacts the material
